# Bioremoval of heavy metals from aqueous solution using dead biomass of indigenous fungi derived from fertilizer industry effluents: isotherm models evaluation and batch optimization

**DOI:** 10.1007/s10534-023-00520-x

**Published:** 2023-07-10

**Authors:** Mervat Morsy Abass Ahmed El-Gendy, Shimaa M. Abdel-Moniem, Nabila S. Ammar, Ahmed Mohamed Ahmed El-Bondkly

**Affiliations:** 1 Chemistry of Natural and Microbial Products Department, National Research Centre, Dokki, 12622 Giza Egypt; 2 Water Pollution Research Department, National Research Centre, El-Buhouth St., Dokki, 12622 Giza Egypt; 3 Genetics and Cytology Department, National Research Centre, Dokki, 12622 Giza Egypt

**Keywords:** Industrial wastewater, *Cladosporium* biomass, Heavy metals, Optimization, Isotherm models, SEM–EDX and FTIR

## Abstract

The present work investigated the utilization of dead biomass of the highly multi-heavy metals tolerant indigenous fungal strain NRCA8 isolated from the mycobiome of fertilizer industry effluents that containing multiple heavy metal ions at high levels to remove Pb^2+^, Ni^2+^, Zn^2+^, and Mn^2+^ as multiple solutes from multi-metals aqueous solutions for the first time. Based on morphotype, lipotype and genotype characteristics, NRCA8 was identified as *Cladosporium* sp. NRCA8. The optimal conditions for the bioremoval procedure in the batch system were pH 5.5 for maximum removal (91.30%, 43.25%, and 41.50%) of Pb^2+^, Zn^2+^ and Mn^2+^ but pH 6.0 supported the maximum bioremoval and uptake of Ni^2+^ (51.60% and 2.42 mg/g) by NRCA8 dead biomass from the multi-metals aqueous solution, respectively. The 30 min run time supported the highest removal efficiency and uptake capacity of all heavy metals under study. Moreover, the equilibrium between the sorbent NRCA8 fungal biomass and sorbates Ni^2+^, Pb^2+^ and Zn^2+^ was attained after increasing the dead biomass dose to 5.0 g/L. Dead NRCA8 biomass was described by scanning electron microscopy, energy-dispersive X-ray spectroscopy and Fourier transform infrared spectrometer before and after biosorption of Pb^2+^, Ni^2+^, Zn^2+^ and Mn^2+^ under multiple metals system. The Langmuir, Freundlich and Dubinin-Kaganer-Radushkevich isotherms were applied to characterize the adsorption equilibrium between Pb^2+^, Ni^2+^, Mn^2+^ and Zn^2+^ and the adsorbent NRCA8. By comparing the obtained coefficient of regression (R^2^) by Freundlich (0.997, 0.723, 0.999, and 0.917), Langmiur (0.974, 0.999, 0.974, and 0.911) and Dubinin-Radushkevich (0.9995, 0.756, 0.9996 and 0.900) isotherms values for Pb^2+^, Zn^2+^, Ni^2+^ and Mn^2+^ adsorption, respectively, it was found that the isotherms are proper in their own merits in characterization the possible of NRCA8 for removal of Pb^2+^, Zn^2+^, Ni^2+^ and Mn^2+^. DKR isotherm is the best for Pb^2+^ and Ni^2+^ (0.9995 and 0.9996) while Langmiur isotherm giving a good fit to the Zn^2+^ sorption (0.9990) as well as Freundlich isotherm giving a good fit to the Mn^2+^ sorption (0.9170). The efficiencies of *Cladosporium* sp. NRCA8 dead biomass for bioremoval of heavy metals from real wastewater under the optimized conditions were Pb^2+^, Ag^+^, Mn^2+^, Zn^2+^ and Al^3+^ ˃ Ni^2+^ ˃ Cr^6+^ ˃ Co^2+^ ˃ Fe^3+^ ˃ Cu^2+^ ˃ Cd^2+^. Dead NRCA8 biomass showed efficient ability to adsorb and reduce harmful components in the industrial effluents to a level acceptable for discharge into the environment.

## Introduction

Overpopulation causes rapid industrialization and thus augmented production of industrial waste. These industrial wastes cause great environmental destruction by contaminating water, air and soil (Ahmed et al. 2021). Industrial effluents from agrochemical industries such as fertilizers, pesticides, and herbicide manufacturing contain high levels of non-biodegradable pollutants such as heavy metals that can be toxic, reactive, carcinogenic or flammable (Ab Rhaman et al. 2022; Santos et al. 2021). Hence, without appropriate dealing and managing approaches, the discharge of industrial wastewater into water bodies can have appalling environmental and health impacts (Duque et al. 2021; El-Gendy et al. 2011, 2017a). Heavy metals including Pb^2+^, Ni^2+^, Mn^2+^, Zn^2+^, Cr^6+^, Cd^2+^, As^3+^ Fe^3+^, Co^2+^, Cu^2+^, and others are non-biodegradable (Razzak et al. 2022; El-Bondkly and El-Gendy 2022; AL-Huqail and El-Bondkly 2022).

Several technologies have been developed to remove heavy metals from aqueous systems (Sumalatha et al. 2023a). Among the various physical, chemical and biological procedures used to treat industrial effluents loaded with metals them bioadsorption has been documented as a promising biotechnology (El-Gendy and El-Bondkly 2016). Biosorption can be defined as a fast process independent of energy on which biological materials or biopolymers acting as sorbents to remove pollutants, such as heavy metals from wastewater through metabolically mediated or physico-chemical pathways of uptake (Sumalatha et al. 2022a, b; Khan et al. 2022). Biosorption has many advantages, such as low cost removing contaminants even in dilute concentrations, using of biomass for removal of heavy metals, cheaper production of biomass (live and dead bacteria or fungi) can be used as biosorbents for the process of biosorption, multiple heavy metals uptake at a time, treatment of large volume of effluents, no necessity for chemical additions as extremely selective for uptake and removal of specific metals and it have great potential to be an economic method for heavy metals removal from industrial effluent (Khan et al. 2022; El-Gendy et al. 2011, 2017a, b). Bioadsorption of adsorbates from the aqueous solutions to the surface of the fungal biomass as green sorbent has advantages as small handling time, slight space requisite, low energy and chemical consumptions, low-cost and effective environmentally friendly technolog (Alzahrani et al. 2017; Alzahrani and El-Gendy 2019).

In dematiaceous hyphomycetes, *Cladosporium* is the largest of the genera, and its species have been described as being among the most common fungi in both indoor and outdoor environments (Becchimanzi et al. 2021; El-Gendy et al. 2017b). Previous works proved that the dead fungal biomass could be an efficient option to reduce various toxic compounds and heavy metals from industrial wastewater with great adsorption capacity because it's big specific external area and effective surface interaction along with its ability endure temperature difference (Paria et al. 2022; Ab Rhaman et al. 2022). Hence, the goal of this search was to investigation the potential applies of dead fungal biomass as adsorbent for Pb^2+^, Ni^2+^, Mn^2+^, and Zn^2+^ from aqueous solutions, optimization of the batch adsorption operating conditions under multi-metals system including pH, adsorption time, biomass dosage and initial metals ions concentration. Furthermore, the Langmuir, Freundlich and Dubinin-Kaganer-Radushkevich (DKR) isotherm systems were applied to appropriate the equilibrium isotherm of these heavy metals on the surface of the fungal strain NRCA8 under the optimized conditions.

## Materials and methods

### Metal ions solution and factory effluents preparation

Multi-metals ions solution stock composed of Pb^2+^ [Pb (CH_3_COO)_2_], Ni^2+^ (NiCl_2_.6H_2_O), Mn^2+^ (MnSO_4_·H_2_O), and Zn^2+^ [Zn(CH_3_CO_2_)_2_] was prepare by dissolving a proper amount of each metal in deionized water to prepare a concentration of 1000 mg/L. The preferred concentrations of C_o_ = 10, 25, 50, and 100 mg/L of each ion in the multi-metals solution were prepared by dilution of the stock solution. pH was from 2.0 to 6.0 by 0.1 M HCl and 0.1 M NaOH solutions. Real wastewater samples belonging to fertilizer industry were collected from the drainage areas of fertilizer industry wastewater at the Manqabad industrial regions of Assiut, Egypt in poly ethylene bottles of 1 L. The wastewater samples were gathered, filtered and divided into three portions. The first was processed immediately for the isolation of their fungal mycobiome; the second was located in sterile flasks including 2.5 mL nitric acid and stay in at 4 °C until analyses for their characteristics before treatment within 24 h of collection by using Agilent 5100 Synchronous Vertical Dual View (SVDV) ICP-OES, with Agilent Vapor Generation Accessory VGA 77. The third kept in − 80 °C until treatment with the dead biomass of selected fungus based on the optimization experiments followed by analyses for its Pb^2+^, Ni^2+^, Mn^2+^, and Zn^2+^ contents and other properties.

### Isolation of mycobiome from the fertilizer industrial effluents

The fungal biosorbents were isolated from fertilizer manufacturing industrial wastewater (drainage areas, Manqabad, Assiut, Egypt). Contaminated water drainage samples were filtered, serially diluted using the serial dilution technique and inoculated into potato dextrose agar (PDA) medium. The plates were incubated for 10 days at 28 °C. The hyper multi-metals tolerant isolate NRCA8 was selected, identified and analyzed for its biosorption efficiency and uptake capacity of different heavy metals from the multi-metals aqueous solutions as previously described. More over the ability of this strain to enhance the industrial wastewater properties was evaluated under the conditions optimized earlier in the batch process including pH, contact time, biomass dosages and initial heavy metals concentrations.

### Screening of fungal strains for the higher multi-metals tolerant strain under single and multi-metals types

Different concentrations of Pb^2+^, Ni^2+^, Mn^2+^, and Zn^2+^ were prepared individually and in different combinations of binary, tertiary and quartet of these metals in a series increasing concentrations of these heavy metals from 0.1 to 6.0 g/L in equal mass ratios. These concentrations of each metal added to the Czapek yeast extract agar (CYEA) medium, individually to investigate the resistance of fungal mycobiome of fertilizer industrial wastewater and selected the hyper multi-metals tolerant strain. All experiments were conducted in duplicate. The inoculated plates were incubated at 30 °C for 7 days to confirm their growth at these varied metals concentrations individually and in mixtures. Growth test was conducted and calculated the higher tolerance concentration for each treatment. The strain which showed the higher tolerance concentrations under single, binary and multi-metals conditions was chosen for the further study.

### Description and identification of the excessive tolerant isolate NRCA8

Description and identification of the strain under study NRCA8 was performed based on its morphotype, lipotype and genotype as earlier described (Crous et al. 2009; Domsch et al. 1980; Kujur and Patel 2014; Ogórek et al. 2012; Schubert et al. 2007; Stahl and Klug 1996; Weete 1980). The fungus morphology and colony characteristics on PDA, malt extract agar (MEA) and oat agar (OA) media (Difco Laboratories, USA) were recorded and photographed after 14 days of incubation at 25 °C. The surface and reverse colors were rated using the charts of Rayner (1970). For micro-morphological observation, preparations of colonies made on PDA were mounted onto Shear's solution and conidial development and branching patterns were studies (Crous et al. 2009; Schubert et al. 2007). Temperature range for the growth of fungal isolate was established on PDA cultures after 14 days at temperatures ranging from 5 to 40 °C at intervals of 5 °C. Also pH range for growth was determined by culturing NRCA8 at pH ranged from 3.0 to 11.0 on PDA.

### Molecular identification of the hyper tolerant isolate NRCA8

DNA was extracted and purified from culture NRCA8 growing on PDA after 7 days of incubation at 28 °C, the DNA of isolate used as the template for the PCR, for isolate NRCA8, partial sequence of the rDNA with primers ITS1 and ITS4 was amplified for the internal transcribed spacer (ITS) region of rDNA, amplification, PCR product purification and sequencing in both directions were performed following the previous protocol (El-Bondkly 2012; El-Gendy et al. 2018; El-Bondkly and El-Gendy 2022; White et al. 1990). Finally, the software SeqMan (DNAStarLasergene) was applied. The sequence achieved was matched with other fungal sequences put in the NCBI database using the BLASTn. Phylogenetic reconstruction was made with the phylogenetic marker (ITS) approved for perfect identification at the species level using maximum likelihood (ML) analyses, with the MEGA11 software (Kumar et al. 2018; Tamura et al. 2011, 2021).

### The dead fungal biomass (adsorbent) preparation

The dead biomass of the NRCA8 isolate was used as natural biosorbent for Pb^2+^, Ni^2+^, Mn^2+^, and Zn^2+^ from aqueous solutions under multi-metals system. Ten-days old culture spores (10^6^ spore/mL) were transferred individually into 500 mL Erlenmeyer flasks, each including 100 mL potato dextrose broth medium and incubated at 30 °C, 150 rpm on a rotary shaker for 10 days. The biomass of isolate was pelletized by filtration using filter papers (Whatman No. 1), washed with 0.1 M NaCl followed by deionized water. Dead biomass was obtained by autoclaving, washed with 0.1 M NaCl, pre weighted and dried in an oven at 60 °C followed by crushing to a fine powder and kept in sterile polyethylene bottles at 4 °C until use.

### Evaluation of adsorption performance of the selected fungal isolate NRCA8

The biosorption trials were conducted under multi-metals system in quick-fit flasks containing biosorbent dosage 1 g/L of the dead biomass in working volume 50 mL aliquots of a mixture of Pb^2+^, Ni^2+^, Mn^2+^, and Zn^2+^ at a concentration of 100 mg/L (25 mg/L for each). Flasks were kept on rotary shakers (150 rpm) at 30 °C and pH 5.0 for 30 min. The samples were filtrated and the concentration of each metal ion in the multi-metals solution was estimated. All samples were digested using Anton-Paar microwave digestion method (APHA 2017). The supernatants were analyzed for residual heavy metals. Heavy metal solutions without biomass were assisted as control, trials were conducted in duplicate and average values were calculated. The data was recorded in percentage using the following equations: the metal removal efficiency percentage (R%) was given in the multi-metals solution of Pb^2+^, Ni^2+^, Mn^2+^, and Zn^2+^ or in real wastewater according to the equation (Li et al. 2009; Singh et al. 2007):

1 $$ {\text{R = }}\frac{{C_{o} - C_{f} }}{{C_{o} }} \times 100{\text{ \% }} $$

where, R is the biosorption efficiencies percentage (%) of each metal separately; C_o_ is the initial metal concentration (mg/L), and C_f_ is the equilibrium or final concentration for each metal calculated separately. Moreover, the adsorption capacity of fungal biomass was assessed by the equation:

2 $$ {\text{q}}_{{\text{t}}} = \left( {{\text{C}}_{{\text{o}}} - {\text{C}}_{{\text{t}}} } \right) \times \frac{{\text{V}}}{{\text{m}}} $$

wherever q_t_ is adsorption capacity (mg/g) and *C*_*t*_ is metal concentration (mg/L) at time = t (min), V is the solution volume (L), and m is adsorbent mass (g).

### Optimization of the biosorption batch factors

Batch trials were carried out in 250 mL Erlenmeyer flasks containing multi-metals solution of Pb^2+^, Ni^2+^, Mn^2+^, and Zn^2+^ solutes, following optimization process. The impact of pH on metal biosorption by NRCA8 biomass was evaluated by varying the initial solution pH (2, 4, 5, 5.5, and 6) by dilute HCl or NaOH, the pH values of the experiment are not set higher than 6 for avoiding any metal precipitation as hydroxide as well as the effect of the adsorption time was evaluated at various contact times (10, 20, 30, 60, 90, and 180 min) at the optimum pH. Moreover, the effects of the dead fungal biomass dosage (1, 2, 5, and 10 mg/mL) was evaluated at the optimum pH and contact time. The initial concentration of Pb^2+^, Ni^2+^, Mn^2+^, and Zn^2+^ metals (C_o_ = 10, 25, 50, and 100 mg/L of each metal in the mixture) were evaluated at the proceed optimum conditions. In each experiment, flasks were permitted to equilibrate on a rotating shaker and samples were collected after the appropriate period, aqueous solutions were filtered and each filtrat analyzed for residual concentration of each metal as well as its biosorption efficiency (%) and uptake (mg/g) were quantified to determine the optimum process parameters for maximum metal ion biosorption.

### Adsorption isotherm

The uptake of heavy metal ions by the fungal biomass was evaluated by the various adsorption isotherm systems containing Langmuir, Freundlich and Dubinin–Kaganer–Radushkevich (DKR) models. The linear form of Langmuir equation is characterized by the following equation:

3 $$ \frac{Ce}{{Qe}} = \frac{1}{{Qmax{ }KL}} + \frac{Ce}{{Qmax{ }}} $$

where *Q*_*e*_ is the equilibrium uptake capacity (mg/g), *C*_*e*_ is the concentration of adsorbate molecule remaining in solution at equilibrium (mg/L), *Q*_*max*_ is the maximum ions uptake per unit mass of fungi, (mg/g) related to adsorption capacity that represents monolayer coverage and *K*_*L*_ is the Langmuir constant equivalent to the enthalpy of adsorption (L/mg). Therefore, the linear plot of *Ce*/*qe* versus *Ce* gives a straight line of slope 1/*q*max and intercepts 1/(*q*maxK_L_) (Dąbrowski 2001). The Langmuir system were applied to determine the separation factor RL, as stated by Eq. (4) according to (Fawzy et al. 2018).

4 $$ {\text{RL = }}\frac{1}{{1 + K_{l} C_{o} }} $$

The Freundlich isotherm system suggests the heterogeneous adsorption of the surface that has unequal available sites. The linear equation can be written as follows:

5 $$ {\text{InQe = InKf }} + \frac{1}{n} In{\text{Ce }} $$

*Q*_*e*_ (mg/g) is the amount of metal ion adsorbed on adsorbent at equilibrium, *C*_*e*_ (mg/L) the equilibrium concentration of metal ion in the solution, *K*_*f*_ (mg^1−1/n^ L^1/n^ g^−1^) is a Freundlich isotherm constant describing the adsorption capacity and n is empirical parameter related with multiple layer coverage (Ayawei et al. 2017; Hamdaoui and Naffrechoux 2007).

The results were also fitted with DKR isotherm model to estimate the nature of sorption process as chemical or physical and estimate the mean energy of sorption. The linear equation of DKR isotherm is

6 $$ {\text{qe = qm}}exp - \beta \varepsilon 2 $$

where *q*_*e*_ is the number of metal ion adsorbed per unit weight of adsorbent (mol/g), *q*_*m*_ is the maximum sorption capacity, b is the activity coefficient related to mean sorption energy, and Ɛ is the Polanyi potential, which is equal to:

7 $$ \varepsilon {\text{ = RTIn}} \left( {1 + {\raise0.7ex\hbox{$1$} \!\mathord{\left/ {\vphantom {1 {Ce}}}\right.\kern-0pt} \!\lower0.7ex\hbox{${Ce}$}}} \right) $$

where R is the gas constant (kJ/kmol K) and T is the temperature (K). By plotting a relationship between lnq_e_ and ε^2^, β and q_DR_ can be obtained. (D-R) isotherm parameter β used to determine adsorption energy E (KJ/mol) as follows:

8 $$ E\;{ = }\;\frac{1}{{\sqrt { - 2\beta } }} $$

### Scanning electron microscope (SEM), energy-dispersive X-ray spectroscopy (EDX) and Fourier transform infrared spectroscopy (FTIR) analyses

SEM–EDX method was applied to define the chemical description of fungal biomass before and after adsorption of heavy metal ions Pb^2+^, Zn^2+^, Ni^2+^ and Mn^2+^. The fungal biomass amended with multi-metals solution of these ions at initial concentration of 100 mg/L of each was used for SEM analysis (SEM Quanta FEG 250 with field emission gun, FEI Company—Netherlands) at the Central Laboratory (National Research Centre, Egypt). The functional groups on the biomass surface were determined by a Fourier transform infrared spectrometer (Broker Vertex80v, Germany). Confirmation of presence of metal ions on the fungal biomass surface was tested using EDX analysis using an X-ray micro-analyzer connected to a scanning electron microscope. The individual ratios given represent the average of ten measurements. In FT-IR were used to measure the transmittance spectra recorded in the range of 4000–400 cm^−1^ with resolution 4 cm^−1^ at the Central Laboratory of National Research Centre, Egypt to define the vibration frequency groups in the biosorbent NRCA8 before and after biosorption of heavy metals under study from the multi-metals solution.

### Wastewater analyses

For the analyses of wastewater, samples collected from the fertilizer industry effluents, Mankapad, Assuit, Egypt were exposed to centrifugation at 2000 rpm for 2 min, filtration by Whatman filter paper with 0.2 μm pore size, acid digestion according to APHA (2017) followed by determination the initial and final concentrations of metal ions concentrations before and after treatment with NRCA8. Moreover, the other parameters including total suspended solids (TSS), total dissolved solids (TDS), oil and grease, chemical oxygen demand (COD), nitrogen (N), and phosphorus (P) were evaluated following the standard approaches for the analysis of water and wastewater (APHA 2017).

### Statistical analysis

The data was statistically processed by analyzes of variance (ANOVA), followed by Tukey’s tests when significant effects were detected (P ≤ 0.05). Data were expressed as means ± standard error.

## Results and discussions

### Selection of the high metals tolerant fungal strain

The mycobiome derived from fertilizer industrial effluents were evaluated and screened for their tolerance of Ni^2+^, Pb^2+^, Mn^2+^ and Zn^2+^ in single and multimetals systems, among them the strain under the isolation code NRCA8 showed the highest metals tolerant behavior against these heavy metals (Table 1). Data in Table 1 indicated that the highest concentrations of Pb^2+^, Ni^2+^, Zn^2+^ and Mn^2+^ that the strain was able to grow were 5.0, 2.5, 3.2, and 2.6 g/L in the presence of single metal in growth medium while it was estimated at 5.4, 6.0, 5.5, 2.7, 2.5, and 2.7 g/L in the presence of the binary combinations (Pb^2+^ + Ni^2+^), (Pb^2+^ + Zn^2+^), (Pb^2+^ + Mn^2+^), (Ni^2+^ + Zn^2+^), (Ni^2+^ + Mn^2+^) and (Zn^2+^ + Mn^2+^), respectively (Table 1). Moreover, in the multi-metals system in the growth medium composed of (Pb^2+^ + Ni^2+^  + Zn^2+^), (Pb^2+^ + Ni^2+^ + Mn^2+^), (Pb^2+^ + Zn^2+^  + Mn^2+^), (Ni^2+^ + Mn^2+^ + Zn^2+^) and (Pb^2+^ + Ni^2+^ + Zn^2+^ + Mn^2+^), individually the highest initial multimetals concentration of each ion the strain was able to grow at 4.1, 4.0, 5.0, 3.45, and 4.0 g/L, respectively (Table 1). Then the fungal strain that showed high adaptive tolerance to Ni^2+^, Pb^2+^, Mn^2+^ and Zn^2+^ under single, binary, ternary and quaternary metals systems was then selected for further studies. Strain NRCA8 has created exceptional performance and can reduce concentrations of Ni^2+^, Pb^2+^, Mn^2+^ and Zn^2+^ well below the irrigation threshold level set by the Food and Agriculture Organization (FAO). In agreement with our results Madhuri et al. (2022) who stated that fungi are help maintain tolerance to heavy metals in various contaminated sites by developing different methods of resistance against different heavy metals, and have the potential to survive through adapting or mutating at high concentrations of heavy metals as well as can also decrease heavy metals from environment to some extent, and thus these approaches would facilitate the development of enhanced methods for the bioremediation of heavy metals in the environment. Furthermore, Dey et al. (2016) reported that the fungal isolates *A.* *terreus* AML02, *Paecilomyces* *fumosoroseus* 4099*,* *Beauveria* *bassiana* 4580, *A.* *terreus* PD-17, and *A.* *fumigatus* PD-18 exposed to a mixture of multiple metals (Cd, Cr, Cu, Ni, Pb and Zn) of different concentrations (6, 12, 18, and 30 mg/L) showed high metals tolerance index for each metal in the mixture suggesting their better adaptability to multi-metals stress.

**Table 1:** Screening of fungal isolates obtained from the microbiome of the fertilizer industrial wastewater for the tolerance concentrations of Pb^2+^, Ni^2+^, Zn^2+^ and Mn^2+^ in single, binary, ternary and quarterly systems

Fungal isolates code	The maximum tolerance of each heavy metals concentration (g/L)														
	Single system				Binary system						Ternary system				Quarterly system
	Pb^2+^	Ni^2+^	Zn^2+^	Mn^2+^	Pb^2+^ + Ni^2+^	Pb^2+^ + Zn^2+^	Pb^2+^ + Mn^2+^	Ni^2+^ + Zn^2+^	Ni^2+^ + Mn^2+^	Zn^2+^ + Mn^2+^	Pb^2+^ + Ni^2+^ + Zn^2+^	Pb^2+^ + Ni^2+^ + Mn^2+^	Pb^2+^ + Zn^2+^ + Mn^2+^	Ni^2+^ + Mn^2+^ + Zn^2+^	Pb^2+^ + Ni^2+^ + Zn^2+^ + Mn^2+^
NRCA1	1.00	0.3	0.60	0.20	0.40	1.50	0.25	0.50	0.20	0.30	0.20	0.25	0.20	0.20	0.15
NRCA2	0.80	0.5	1.50	0.60	0.45	1.00	0.50	0.65	0.50	0.85	0.70	0.50	0.65	0.70	0.60
NRCA3	1/00	1.6	1.00	1.50	1.00	1.00	2.00	1.00	1.70	1.00	1.20	1.40	1.00	1.20	0.95
NRCA4	2.00	1.0	0.80	0.80	1.40	0.90	1.00	0.90	0.85	0.75	1.10	1.00	1.00	1.00	1.10
NRCA5	1.00	0.25	1.20	0.50	0.40	1.00	0.60	0.50	0.25	0.60	0.70	0.55	0.80	0.60	0.70
NRCA6	0.50	0.20	0.90	0.40	0.30	0.65	0.50	0.30	0.25	0.45	0.35	0.30	0.50	0.33	0.50
NRCA7	0.50	0.20	0.50	0.20	0.23	0.40	0.25	0.30	0.15	0.33	0.38	0.25	0.35	0.23	0.40
NRCA8	5.00	2.50	3.20	2.60	5.40	6.00	5.50	2.70	2.50	2.70	4.10	4.00	5.00	3.45	4.00
NRCA9	2.50	1.00	1.80	0.90	2.00	2.20	1.34	1.48	0.88	1.29	1.27	1.00	1.36	1.04	1.39
NRCA10	0.60	0.90	0.20	0.10	0.64	0.49	0.15	0.26	0.10	0.18	0.39	0.34	0.20	0.40	0.40
NRCA11	0.90	0.60	1.60	0.40	0.82	1.30	0.75	1.00	0.34	0.90	1.00	0.86	1.15	0.85	1.00
NRCA12	2.50	0.90	0.40	0.70	1.62	1.00	1.40	0.69	1.24	0.56	1.57	1.70	1.50	0.89	1.61
NRCA13	2.00	0.40	0.65	1.00	1.10	1.59	1.60	0.59	0.62	1.00	0.63	1.00	1.00	0.90	0.80
NRCA14	1.60	1.00	1.00	1.00	1.30	1.36	1.29	1.00	1.00	1.00	1.30	1.38	1.20	1.00	1.10
NRCA15	0.40	0.20	0.60	0.50	0.32	0.39	0.40	0.16	0.46	0.50	0.30	0.25	0.52	0.39	0.40
NRCA16	0.50	0.60	1.10	1.80	0.42	0.58	0.60	0.74	0.85	1.00	0.80	0.86	0.90	1.10	0.92
NRCA17	0.90	0.85	0.80	1.40	0.90	0.90	1.20	0.80	1.00	1.00	0.87	1.00	1.12	1.06	1.10
NRCA18	1.00	1.20	1.00	0.32	1.20	1.00	0.73	1.25	0.69	0.58	1.20	0.50	0.49	0.51	0.73
NRCA19	1.50	0.95	2.40	0.68	1.10	2.00	0.82	1.43	0.75	1.10	1.91	1.00	1.50	1.04	1.67
NRCA20	2.20	0.45	0.60	1.00	1.00	1.25	1.51	0.42	0.65	0.82	1.00	1.36	1.50	0.92	1.40
NRCA21	1.00	1.45	0.40	1.00	1.40	0.63	1.00	0.59	1.18	0.50	0.85	1.10	0.77	0.83	1.00
NRCA22	1.20	0.60	0.50	1.50	0.89	0.68	1.50	0.70	0.84	0.78	0.85	0.90	0.98	0.87	1.15
NRCA23	1.00	1.75	1.60	0.80	1.40	2.17	0.92	2.00	0.99	1.36	1.31	0.93	1.00	1.00	1.00

### Characterization and identification of highest tolerant isolate NRCA8

**Fig. 1:**
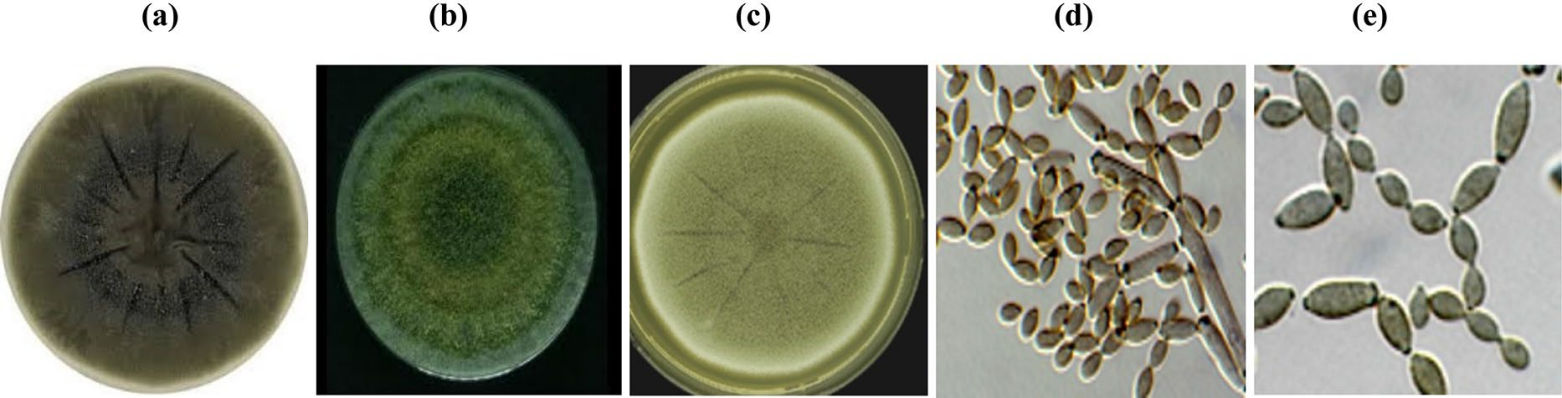
*Cladosporium* sp. NRCA8 colonies on PDA (**a**), MEA (**b**) and OA (**c**) media after 14 days at 28 °C (scale bars; 10 mm). Conidiophores with conidial chains (**d**), ramoconidia and conidia (**e**) (scale bars; 10 μm)

The reproductive structures of the selected strain NRCA8 after 14 days of growth on different media are shown in Table 2 and Fig. 1a–e. The NRCA8 isolate on PDA formed distinct microscopic features including a short cylindrical mycelium, 1.5–4.5 μm wide, olivaceous-brown, smooth or verruculose concerning the base of conidiophores, thick-walls. Conidiophores micronematous to semimacronematous, arising from terminal hyphae, concolourous with hyphae, unbranched, non-nodulose or geniculate, straight, septate, olivaceous brown to pale brown, about 20–120 μm long and 2.5–4.0 μm wide, smooth-walled, slightly thickened and presenting rise to conidiogenous apparatus with chains of branched conidia. Conidiogenous cells integrated, terminal, cylindrical, geniculate at the apex or situated on short lateral outgrowths at the apex in terminal cells, 15–38 × 3–4 μm, bearing single or two conidiogenous loci 0.8–1.5 μm diam, thickened, and slightly darkened and refractive. Ramoconidia subcylindrical to cylindrical, (0–1)-septate, 20–30 × 2–5 μm, smooth pale olivaceous to pale brown (Table 2, Fig. 1d). Conidia forming branched chains in all directions, aseptate, olive to pale brown and smooth; terminal conidia 3–8 in the terminal unbranched part of the chain, small, ellipsoid to obovoid, 3–5 × 1.8–3.5 μm, pale olivaceous-brown or pale brown, walls unthickened, apex rounded and attenuated towards the base but intercalary conidia ellipsoid to fusiform and aseptate with sizes ranging 5.0–11.0 × 2.5–3.5 μm, attenuated towards apex and base with 1.0–3.0 distal hila (Table 2, Fig. 1e). Secondary ramoconidia were smooth pale olivaceous-brown, ellipsoidal or nearly cylindrical, 0–1-septate, (8.0–17.0 × 2.5–4.0 μm), and walls unthickened with 2–4 distal hila (Table 2, Fig. 1e).

**Table 2:** Morphological, microscopic, physiological and biochemical characteristics of NRCA8 isolate

Morphotypic and lipotypic characteristics after 14 days incubation period												
**Macroscopic morphology**					**Microscopic morphology**							
**PDA** ***Colonies*** ***Margins*** ***Mycelium*** **MEA** ***Colonies*** ***Margins*** ***Mycelium*** **OA** ***Colonies*** ***Margins*** ***Mycelium***	Attaining 38–69 mm after 14 days, iron-grey to olivaceous-grey, reverse grey to olivaceous-black, velvety to fluffy Margins regular, narrow, even and smooth, smoke gray Aerial mycelium sparse, diffuse to more intense in some spots, more abundantly formed in colony centre, fluffy with numerous small to large prominent exudates, and sporulation profuse Reaching 60–90 mm, grow in successive irregular yellowish green rings forming concentric zones with slightly lobate margins, dark olive green at centre, become slightly heaped and develop gentle folds as it ages Margins are grayish green, wide and irregular Aerial mycellium cottony to fluffy, velvety at the center, growth flat, and reverse side dark blackish green Attaining 40–63 mm olivaceous green to olivaceous because of abundant sporulation, whitish at margins; flat, slightly dusty, and reverse leaden-grey to black Margins regular, narrow to broad, and white Aerial mycelium diffuse without prominent exudates, and sporulation profuse				**Mycelium** **Conidiophores** **Conidiogenous cells** **Ramoconidia** **Conidia** **Terminal conidia** **Intercalary conidia** **Secondary ramoconidia**			Short, cylindrical, 1.5–4.5 μm wide, olivaceous-brown, smooth or verrucose towards the base of conidiophores, and thick-walls Micronematous to semimacronematous, ascending from terminal hyphae, concolourous with hyphae, unbranched, non-nodulose, non-geniculate, straight or slightly verrucose, septate, not restricted, olivaceous brown to pale brown, 20–120 μm long and 2.5–4.0 μm wide, smooth-walled, slightly thickened, and giving rise to conidiogenous apparatus with chains of conidia Integrated, terminal, cylindrical, geniculate at the apex or situated on short lateral outgrowths at the apex in terminal cells, 15–38 × 3–4 μm, bearing single or two conidiogenous loci 0.8–1.5 μm diam, thickened, slightly darkened and refractive Subcylindrical to cylindrical, (0–1)-septate, 20–30 × 2–5 μm, and smooth pale olivaceous to pale brown*.* Conidia making branched chains in all directions, aseptate, olive to pale brown, and smooth to verruculose 3–8 small terminal conidia in the terminal unbranched part of the chain, ellipsoid to obovoid, 3–5 × 1.8–3.5 μm, pale olivaceous-brown or pale brown, walls unthickened, apex rounded and attenuated towards the base Ellipsoid to fusiform, aseptate, 5.0–11.0 × 2.5–3.5 μm, attenuated towards apex and base with 1.0–3.0 distal hila Smooth, pale olivaceous-brown, ellipsoidal or nearly cylindrical, 0–1-septate, 8.0–17.0 × 2.5–4.0 μm, and walls unthickened with 2–4 distal hila				
**Maximum temp**	37 °C				**Maximum pH**			9.5				
**Optimum temp**	25 °C				**Optimum pH**			5.0 – 6.0				
**Minimum temp**	5 °C				**Minimum pH**			4.0				
**Fatty acid**	14:0	14:1	16:0	16:1		18:0	18:1		18:2	20:4	22:6	18:1 ω9c
**(%)**	1.22 ± 0.16	3.19 ± 0.28	24.61 ± 1.39	4.10 ± 0.36		0.67 ± 0.04	22.37 ± 1.45		24.20 ± 1.55	3.40 ± 0.39	10.00 ± 0.84	6.24 ± 0.51

Culture characteristics of isolate NRCA8 were evaluated after 14 days of growth on PDA, MEA and OA (Table 2, Fig. 1a–c). Colonies on PDA attaining 38–69 mm after 14 days, iron-grey to olivaceous-grey, reverse grey to olivaceous-black, velvety to fluffy; margins regular, narrow, even and smooth, smoke gray; aerial mycelium sparse, diffuse to more intense in some spots, more abundantly made in colony center, fluffy with numerous small to large prominent exudates, and sporulation profuse (Table 2, Fig. 1a). Colonies on MEA getting 60–90 mm, growth in successive irregular yellowish green rings forming concentric zones with slightly lobate margins, dark olive green at center and pale green at margins; become slightly heaped and develop gentle folds as it ages, aerial mycelium cottony to fluffy, velvety at the center, growth flat; reverse side dark blackish green; margins are grayish green, wide and irregular (Table 2, Fig. 1b). Colonies on OA were attaining 40–63 mm, olivaceous green to olivaceous due to abundant sporulation, whitish at margins; flat, slightly dusty, reverse leaden-grey to black; margins regular, narrow to broad; white; aerial mycelium diffuse without prominent exudates, sporulation profuse (Table 2, Fig. 1c). The maximum, optimum and minimum growth temperature were 37, 25, and 5 °C while pHs were 9.5, 5.0–6.0, and 4.0, respectively (Table 2). *Cladosporium* is one of the largest and heterogeneous genera of hyphomycetes. To determine the classification of this genus, it is essential to study its morphological and molecular lineaments based on ex-type strains. Species can be recognized by polyphasic methods with morphological and molecular markers (Nam et al. 2015).

On the other hand, isolate NRCA8 included fatty acids of carbon chain lengths extending from 14 to 22. The predominant fatty acids detected in strain NRCA8 were 16:0 (24.61 ± 1.39%), 18:2 (24.20 ± 1.55%), 18:1 (22.37 ± 1.45%) and 22:6 (10.00 ± 0.84%), respectively followed by 18:1 ω9c (6.24 ± 0.51%), 16:1 (4.10 ± 0.36%), 20:4 (3.40 ± 0.39%) and 14:1 (3.19 ± 0.28%) (Table 2), which propose their prospective structural role in cell membranes as earlier described (El-Gendy et al. 2017b). However, fatty acids that characterized less than 1% of the total fatty acid content were 14:0 (1.22 ± 0.16%) and 18:0 (0.67 ± 0.04%) (Table 2). Depending on the morphological, microscopic, physiological and biochemical features, strain NRCA8 was defined as a strain belonging to *Ascomycota*, family; *Davidiellaceae* and genus; *Cladosporium*. Earlier investigates described fatty acid types as a reliable method to illustrate fungal species and strains (Kujur and Patel 2014; Stahl and Klug 1996).

### Molecular identification of highest tolerant isolate NRCA8 by ITS region sequencing

**Fig. 2:**
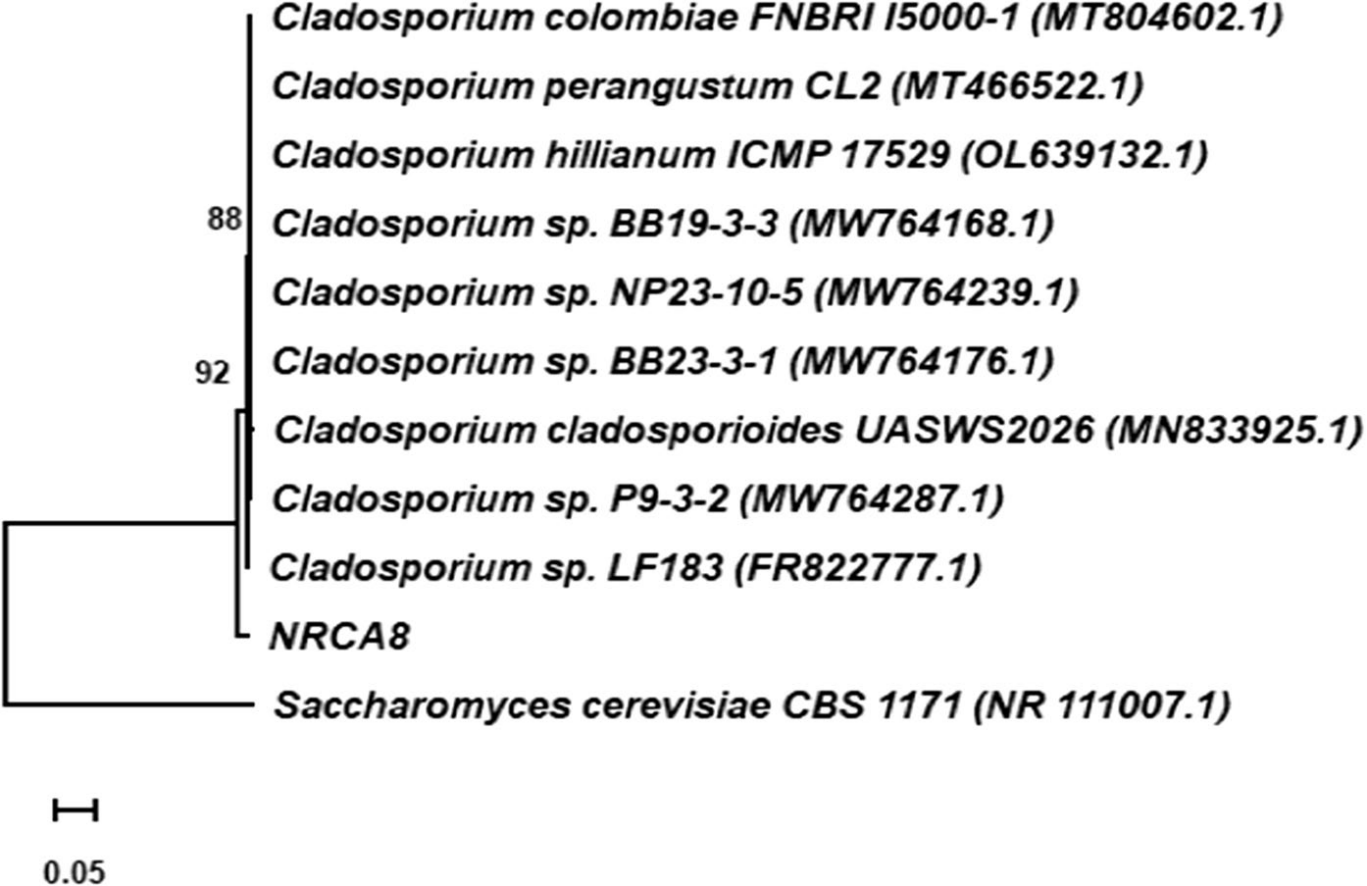
Phylogenetic tree generated by the maximum likelihood based system in the rDNA sequences of the ITS region with isolate NRCA8 belonging to the genus *Cladosporium* obtained through with 1000 repetitions. *Saccharomyces* *cerevisiae* was used as an outgroup

The amplification of the PCR product of the ITS regions of the rDNA of isolated strain NRCA8, generated fragments of 530 bp by ITS1 and ITS4 primers as well as presented to GenBank (accession no. ON667856). The results of the comparisons with Blastn they correspond to a local alignment leading to putative identifications. Therefore, to have greater reliability in the identification, phylogenetic analyzes were performed. A relative analysis by Blastn established that ITS area sequence from isolate NRCA8 had an important identity to the genus *Cladosporium* (Fig. 2). Comparison of isolate NRCA8 with the sequences of reference species in the bank database displayed that isolate NRCA8 exhibited a relationship of 97.90% with *Cladosporium* sp. LF183, and 97.72% with *Cladosporium* sp. NP23-10-5, and *Cladosporium* sp. BB23-3-1 (Fig. 2). Phylogenetic analyzes based on 530 bp were performed by MEGA11, the phylogenetic tree achieved by using the maximum likelihood system is explained in Fig. 2. According to the sequence analysis of the ITS area, along with its phenotypic and chemotypic features, isolate NRCA8 was recognized as *Cladosporium* species and labelled as *Cladosporium* sp. NRCA8. The most prominent markers of fungal phylogenetic are the ITS areas of the rDNA sequences. Previously, the fungal strains NRCF5, Gen 9, Gen 20, and ALAA-20 were identified as *Aspergillus* sp. NRCF5, *Trichoderma* sp. Gen 9, *Cladosporium* sp. Gen 20, and *Fusarium* sp. ALAA-20, respectively based their phenotypic and chemotypic features together with ITS sequences analysis (El-Bondkly 2012; El-Bondkly et al. 2021; El-Gendy et al. 2017b).

### Optimization of operating conditions for different heavy metals removal in multi-metals aqueous solution

**Fig. 3:**
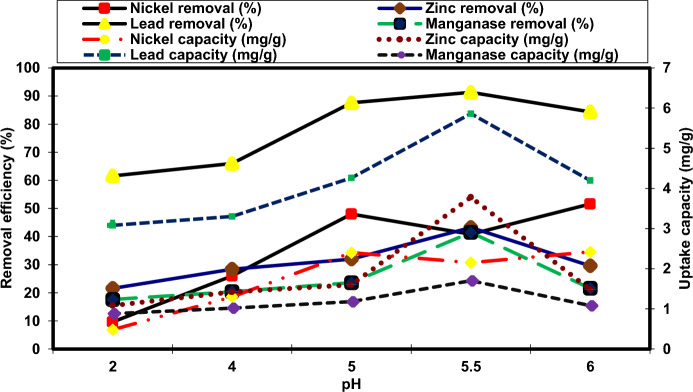
Influence of pH on the bioremoval efficiency and uptake capacity of different heavy metals by the dead biomass of NRCA8 strain

The pH is significant factor that acting an important role in the removal of heavy metals by fungi since it impacts the the metals speciation in the solution and the surface characteristics of the fungi (El-Gendy et al. 2017a). As revealed in Fig. 3, an increase in the pH from 2.0 to 5.5 significantly improved the removal efficiency of Pb^2+^, Zn^2+^ and Mn^2+^ from 61.6, 21.6%, and 17.6 to 91.30%, 43.25, and 41.50%, respectively as well as the uptake of Pb^2+^, Zn^2+^ and Mn^2+^ (5.86, 3.78, and 1.7 mg/g) was attained at pH 5.5, respectively (Fig. 3). This may be attributed to that in a highly acidic environment (pH 2.0), the adsorption locates of NRCA8 became saturated with a positively charged hydrogen ion (H^+^), which could compete with Pb^2+^, Ni^2+^, Mn^2+^ and Zn^2+^ for the active sites that resulted in decrease the heavy metals under study in their aqueous mixture (Fig. 3). With increasing pH, negatively charged OH^−^ tends to dominate the adsorption sites (El-Gendy and El-Bondkly 2016; Fei and Hu 2022). Furthermore, the results in Fig. 3 show that the multi-metals sorption efficiency of Pb^2+^, Zn^2+^ and Mn^2+^ onto NRCA8 doesn’t improved by rising pH value to pH 6.0 and a slightly increase in Ni^2+^ removal (51.60%). Zhang et al. (2020) reported that low pH resulted a negative effect on the adsorption performance because it can accelerate the dissolution/oxidation of functional groups on the surface of the adsorbent, which leads to the release of ions and desorption of the entrapped heavy metals (Table 3).

**Table 3:** Influence of contact time on the bioremoval efficiency (%) and uptake capacity (mg/g) of metal ions (Ni^2+^, Pb^2+^, Mn^2+^ and Zn^2+^) by the dead biomass of the fungus NRCA8

Contact time (min)	Metal ions							
	Mn^2+^		Ni^2+^		Pb^2+^		Zn^2+^	
	Removal (%)	Uptake (mg/g)	Removal (%)	Uptake (mg/g)	Removal (%)	Uptake (mg/g)	Removal (%)	Uptake (mg/g)
10	22.12	1.31	22.13	1.16	71.25	4.56	19.50	2.29
20	25.25	1.49	33.74	1.77	89.50	5.73	24.60	2.89
30	28.81	1.70	40.86	2.15	91.56	5.86	32.17	3.78
60	27.29	1.61	39.67	2.08	90.91	5.82	31.43	3.69
90	25.85	1.53	39.19	2.06	90.39	5.79	31.43	3.69
180	25.17	1.49	39.14	2.06	90.94	5.82	31.13	3.66

Contact time is just one the most important influences affecting biosorption efficiency. The rapid uptake of Mn^2+^, Ni^2+^, Pb^2+^ and Zn^2+^ on NRCA8 (1.70, 2.15, 5.86 and 3.78 mg/g) along with the highest removal efficiency (28.81%, 40.86%, 91.56% and 32.17), respectively were achieved and equilibrium was practically reached after 30 min contact time. The rapid adsorption capability through the initial phase is likely because of the abundance of vacant active locations on the NRCA8 dead biomass and the high concentration gradient of solutes Pb^2+^, Ni^2+^, Zn^2+^ and Mn^2+^ in the multi-metals solution as previously reported for the nickel, zinc and mercury biosorption by dead biomass of metal tolerant fungi (Alzahrani et al. 2017; Alzahrani and El-Gendy 2019). There was no significant change in equilibrium concentration for Pb^2+^, Ni^2+^ and Zn^2+^ ions after 30 min but only a slight decrease for Mn^2+^ ion was observed, these decreases may be attributed to the agglomeration of Pb^2+^, Ni^2+^, Zn^2+^ and Mn^2+^ onto the NRCA8 active sites, the difficulty of occupying the remaining binding locations because of forces concerning the solute molecules that solid and bulk stages and permanent interaction can occur. Then Pb^2+^, Ni^2+^, Zn^2+^ and Mn^2+^ ions successfully diffuse from the boundary layer surrounding the NRCA8 particles to the bulk solution as previously reported (Alothman et al. 2020; Chen et al. 2019; Kumar et al. 2022).

In this research, it was detected that the equilibrium between the sorbent NRCA8 biomass and sorbates Ni^2+^, and Pb^2+^ in the multimetals solution was achieved after increasing the biomass dose to 5.0 g/L, where their bioremoval efficiency reached their maximum values (51.6%, and 91.72%, respectively); afterwards a non-significant decrease in the removal capacity was observed (Table 4). Additionally, the maximum adsorption of Mn^2+^ and Zn^2+^ (47.46% and 43.25%, respectively) were accomplished by further increasing the biosorbent does to 10.0 g/L from the multi-metals aqueous solution. The increased bioremoval efficiency with increasing doses of NRCA8 could be attributed to the larger surface zone of the biosorbent, which in turn increased the availability of active locations for metal ions (Ayele et al. 2021; El-Gendy et al. 2011).

**Table 4:** Influence of biosorbent dose on the bioremoval efficiency (%) and uptake capacity (mg/g) of metal ions (Ni^2+^, Pb^2+^, Mn^2+^ and Zn^2+^) by the dead biomass of the fungus NRCA8

Dose (g/L)	Metal ions							
	Mn^2+^		Ni^2+^		pb^2+^		Zn^2+^	
	Removal (%)	Uptake (mg/g)	Removal (%)	Uptake (mg/g)	Removal (%)	Uptake (mg/g)	Removal (%)	Uptake (mg/g)
1	25.42	3.00	29.52	3.10	89.06	11.40	25.11	5.90
2	28.81	1.70	40.86	2.15	91.56	5.86	32.17	3.78
5	41.53	0.98	51.60	1.24	91.72	2.35	34.89	1.64
10	47.46	0.56	50.48	0.55	89.84	1.15	37.02	0.87

The bioremoval (%) of Pb^2+^, Ni^2+^, Zn^2+^ and Mn^2+^ were evaluated under different initial multi-metals concentrations extending from 10 to 100 mg/L at the optimal operating circumstances for each metal in the multi-metals aqueous solution as shown in Table 5. The improved biosorption at first phases can be accredited to the larger driving force of metal ions into the fungal surface and abundance of vacant binding locations on the biosorbent surface (Alzahrani and El-Gendy 2019; Kumar et al. 2019). The multi-sorption of Pb^2+^, Ni^2+^, Zn^2+^ and Mn^2+^ by NRCA8 dead biomass from the solution was significantly decreased with increasing the early metal concentrations of each metal in the mixture (Table 5), the reduction in heavy metals bioremoval can be associated to the exaggerated quantity of ions about saturation of all available sorption locations on the NRCA8 fungal biomass surface and accomplishment of equilibrium among the sorbent and sorbate, thus averting further adsorption of heavy metal ions (Tu et al. 2018).

**Table 5:** Influence of initial metal concentrations on the bioremoval efficiency (%) and uptake capacity (mg/g) of metal ions (Ni^2+^, Pb^2+^, Mn^2+^ and Zn^2+)^ by the dead biomass of the fungus NRCA8

Initial conc. (mg/L)	Metal ions															
	Mn^2+^				Ni^2+^				Pb^2+^				Zn^2+^			
	Removal (%)	Residual Conc. (mg/L)	Uptake Conc. (mg/L)	Uptake capacity (mg/g)	Removal (%)	Residual Conc. (mg/L)	Uptake Conc. (mg/L)	Uptake capacity (mg/g)	Removal (%)	Residual Conc. (mg/L)	Uptake Conc. (mg/L)	Uptake capacity (mg/g)	Removal (%)	Residual Conc. (mg/L)	Uptake Conc. (mg/L)	Uptake capacity (mg/g)
10	41.50	7.02	4.98	0.99	51.60	5.81	6.19	1.24	91.3	1.04	10.96	2.21	43.25	6.81	5.19	1.04
25	23.60	19.10	5.90	1.18	47.60	13.10	11.90	2.38	87.6	3.10	21.90	4.26	32.00	17.00	8.00	1.60
50	23.20	38.40	11.60	2.32	43.20	28.40	21.60	4.32	80	10.00	40.00	6.40	16.20	41.90	8.10	1.62
100	15.00	85.00	15.00	3.00	38.00	62.00	38.00	7.60	70	30.00	70.00	11.00	8.50	91.50	8.50	1.70

### Evaluation of adsorption isotherm models

Data analysis by isothermal equilibrium adsorption is important for understanding the mechanism of metal ions adsorption into NRCA8. In the current work, the adsorption data between the adsorbate ions (Pb^2+^, Ni^2+^, Mn^2+^ and Zn^2+^) and the adsorbent (dead fungal biomass) at equilibrium condition were evaluated by different models including Langmuir, Freundlich and DKR adsorption isotherms.

**Fig. 4:**
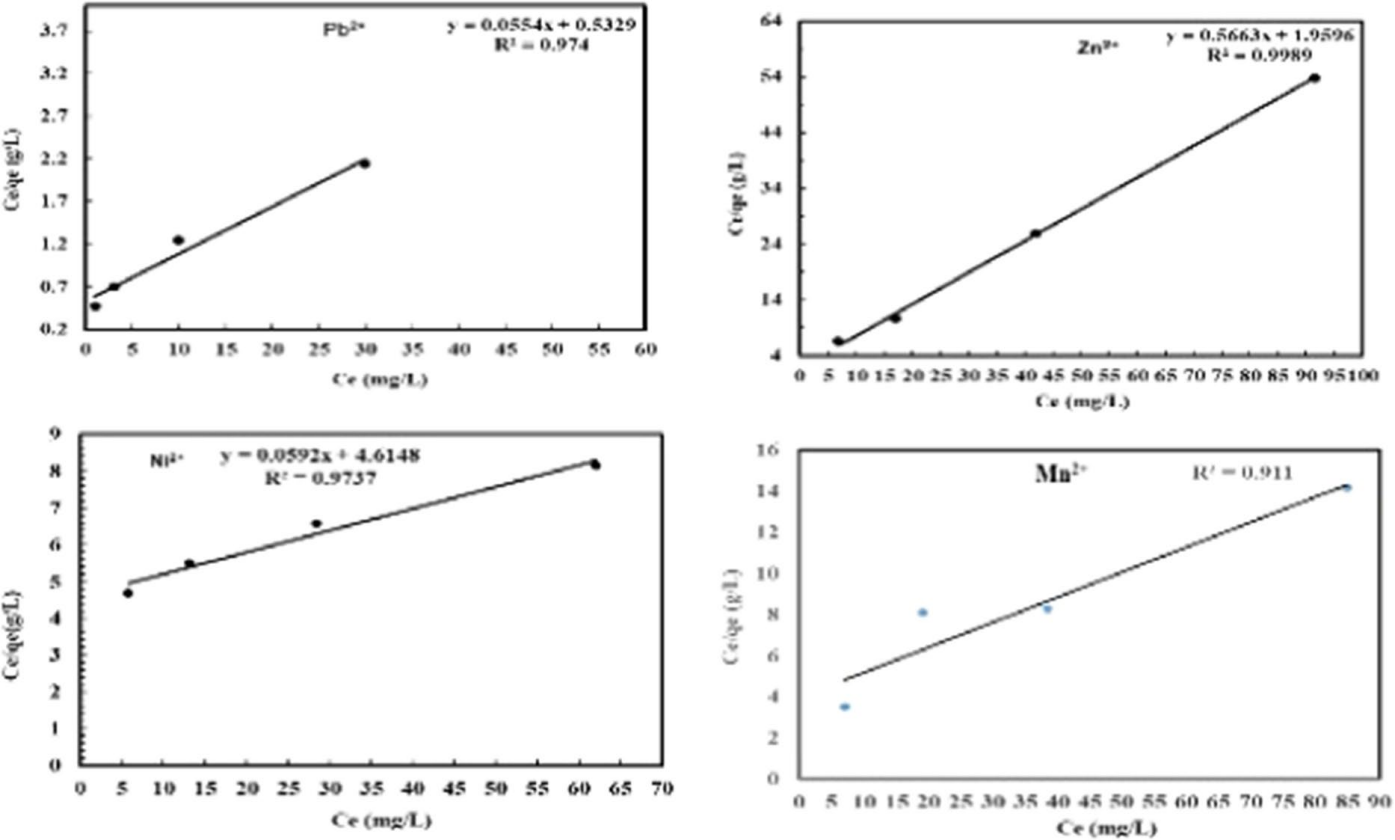
Langmuir isotherm model for Pb^2+^, Zn^2+^, Ni^2+^ and Mn^2+^ adsorption onto dead NRCA8 biomass

The Langmuir isothermal model has been extensively used in adsorption studies. It describes the deposition of adsorbates on the free surface of the adsorbent and the formation of a monolayer adsorbate on the outer surface of the adsorbent. The parameters of the Langmuir isotherm system for Pb^2+^, Zn^2+^, Ni^2+^ and Mn^2+^ adsorption on NRCA8 showed that Q_max_ = 18.05, 1.77, 16.89, and 4.12 mg/g as well as K = 21.54, 18.36, 0.75, and 1.68 L/mg, respectively (Table 6, Fig. 4). Moreover, the determination of the coefficient of the linear equation for Langmuir was R^2^ = 0.974, 0.999, 0.974, and 0.911 for Pb^2+^, Zn^2+^, Ni^2+^ and Mn^2+^ respectively. This noticing indicated that the trial inputs pH, temperature, biomass dosage, contact time and initial metal ions concentration showed positive and linear impacts on the model output beside no over-fitting problem occurred during prediction. Therefore, the Langmuir system can adequately define the adsorption mechanism of Pb^2+^, Zn^2+^, Ni^2+^ and Mn^2+^ onto fungal dead biomass at equilibrium. In the current study the separation factor (RL) for Pb^2+^, Zn^2+^, Ni^2+^ and Mn^2+^ was estimated to be 0.0019, 0.0054, 0.1177, and 0.0562, then the RL values lies in the range of 0 < RL < 1 indicating that adsorption of these heavy metals on surface NRCA8 is preferred at both low and high initial concentration as the RL values are very close to zero. The adsorption is satisfactory at 0 < RL < 1, irreversible at RL = 0, linear at RL = 1, and unfavorable at RL > 1 (Khayyun et al. 2019). Then the Langmiur system provides a good fit to the sorption procedure, which is confirmed by the positive values that gotten for the Langmiur constants presented in Table 6. Similarly, Sumalatha (2023b) proved that Langmuir model and Pseudo second order rate models best suited the adsorption process of heavy metal Cr(VI) from synthetic medium using biodegradable natural polymeric biosorbent, suggesting that ions were adsorbed in monolayer due to their chemical affinity and the thermodynamic characteristics demonstrated the process possibility, spontaneity, and exothermic nature of adsorption.

**Table 6:** Summary of isotherm systems for Pb^2+^, Zn^2+^, Ni^2+^, Pb^2+^ and Mn^2+^ adsorption on dead NRCA8 biomass

Heavy metal	Langmuir			Freundlich			DKR			
	KL (L/mg)	q_max_ (mg/g)	R^2^	K_f_	n	R^2^	Xm (mol/g)	β (mol^2^/j^2^)	E,KJ/mol	R^2^
Pb^2+^	21.54	18.050	0.974	2.235	1.825	0.997	5.140 × 10^−4^	0.424 × 10^−8^	10.860	0.9995
Zn^2+^	18.36	1.766	0.999	0.830	5.740	0.723	4.830 × 10^−5^	0.195 × 10^−8^	15.830	0.7560
Ni^2+^	0.75	16.890	0.974	0.326	1.304	0.999	1.199 × 10^−3^	0.777 × 10^−8^	8.023	0.9996
Mn^2+^	1.68	4.120	0.911	0.207	1.617	0.917	1.910 × 10^−4^	0.499 × 10^−8^	10.012	0.9000

**Fig. 5:**
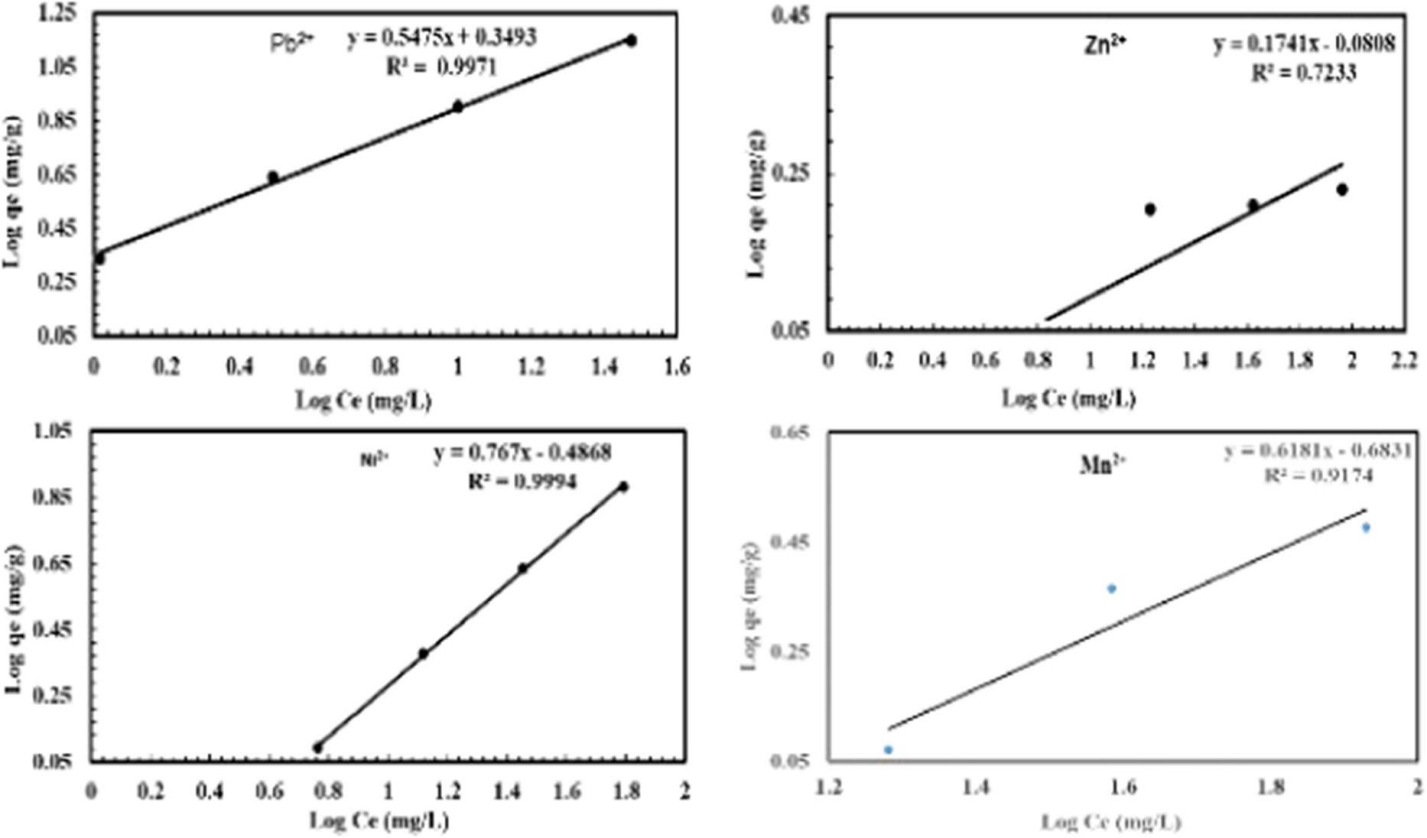
Freundlich isotherm model for Pb^2+^, Zn^2+^, Ni^2+^ and Mn^2+^ adsorption onto dead NRCA8 biomass

From the Freundlich model parameters listed in Table 6 and Fig. 5, n = 1.825, 5.740, 1.304, and 1.617 as well as K_f_ = 2.235, 0.830, 0.326, and 0.207 for Pb^2+^, Zn^2+^, Ni^2+^ and Mn^2+^, respectively. While the regression coefficients for Pb^2+^, Zn^2+^, Ni^2+^ and Mn^2+^ were R^2^ = 0.997, 0.723, 0.999, and 0.917, respectively (Table 6). El-Gendy et al. (2017a) reported that the values of K_f_ and n define the steepness, the isotherm curvature and the adsorption capability of the adsorbents increases at a higher K_f_ value. Khayyun et al. (2019) and El-Gendy et al. (2011, 2017a) described that once 1/n values are in range of 0.1 < 1/n < 1, the adsorption method is desirable, irreversible at 1/n = 0, and unfavorable at 1/n > 1. Hence the 1/n determinants of 0.548, 0.174, 0.767, and 0.618 suggested that the isothermal type desirable, favorable and refer to the strong interaction between fungal biomass and Pb^2+^, Ni^2+^ and Mn^2+^ under multi-metals sorption condition (Table 6, Fig. 5) but it was not much desirable for Zn^2+^ adsorption. In line with our results John et al. (2022) reported that the experimental findings of Pb(II) biosorption from aqueous solutions were fitted with Langmuir, Freundlich and Temkin isotherms but Freundlich model gave good fit with R^2^ = 0.99 and a maximum adsorption capacity of 36.49 mg/g using dried turmeric leaves powder as biosorbent.

**Fig. 6:**
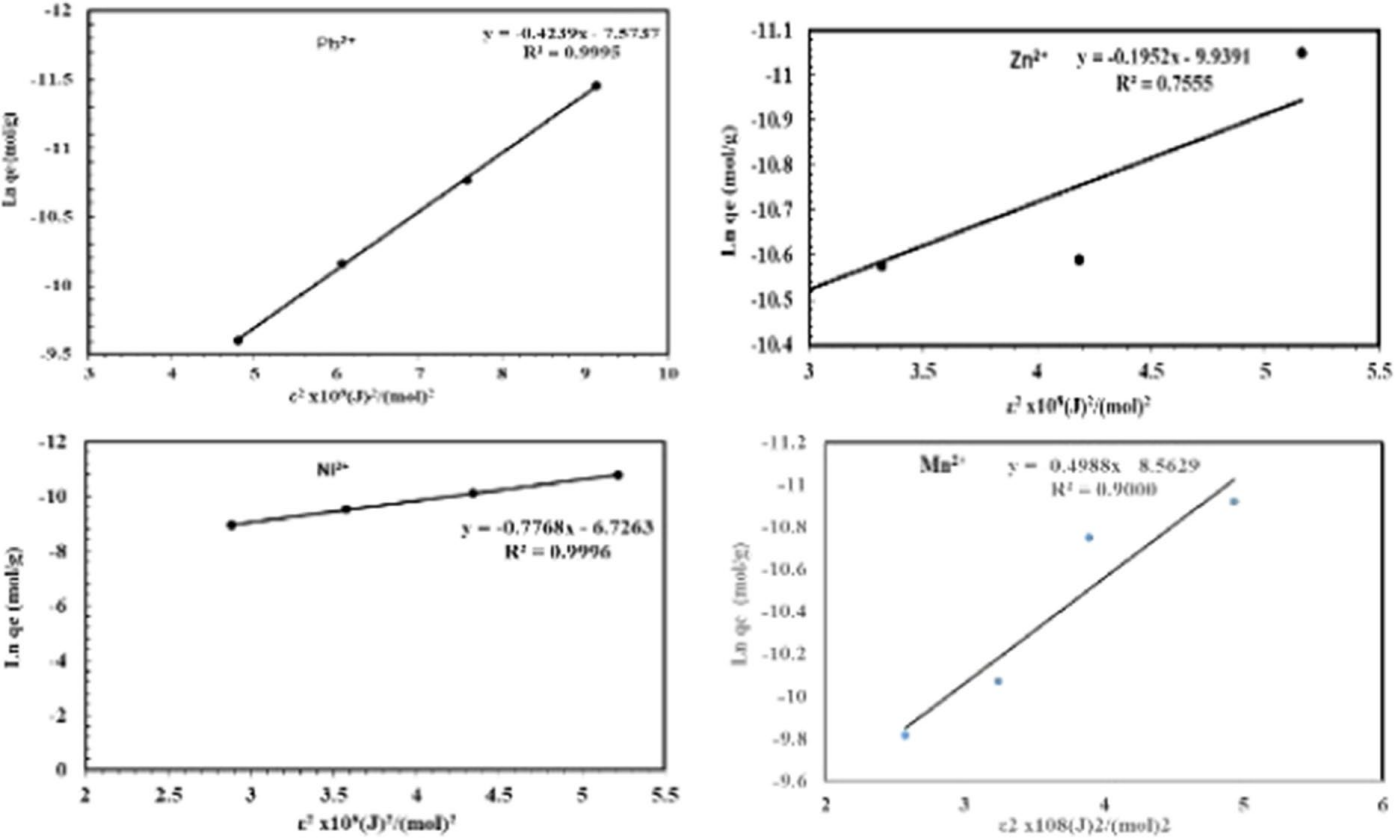
Dubinin–Kaganer–Radushkevich (DKR)isotherm model for Pb^2+^, Zn^2+^, Ni^2+^ and Mn^2+^ adsorption onto dead NRCA8 biomass

The DKR isotherm model in Table 6 and Fig. 6 was selected to assessment the characteristics porosity of the dead biomass and the apparent energy of adsorption as well as describe the equilibrium between the adsorbates and the adsorbent. The maximum sorption capacity (saturation capacity in mol/g), X_m_, and values describing the total specific micropore volume of the sorbent were estimated for Pb^2+^, Zn^2+^, Mn^2+^ and Ni^2+^ (5.14 × 10^−4^, 4.83 × 10^−5^, 1.91 × 10^−4^, and 1.19 × 10^−3^ mol/g), respectively (Table 6). The parameters of the DKR model recorded in Table 6 indicated that the determination of the linear equation coefficient for DKR was R^2^ = 0.9995, 0.7560, 0.9996, and 0.9000 for Pb^2+^, Zn^2+^, Ni^2+^ and Mn^2+^, respectively. The type of adsorption can be predicted by calculating the adsorption energy E, and free energy *E* for Pb^2+^, Zn^2+^, Ni^2+^ and Mn^2+^ (10.86, 15.83, 8.02, and 10.01 kJ/mol) were positive values, which showed the endothermic nature of the heavy metals sorption method by NRCA8 dead biomass. In the present work, the energy values for heavy metals sorption on NRCA8 biomass were between 8 and 16 kJ/mol which indicated that the sorption process can be construed by ion exchange as reported before (Embaby et al. 2021; Igwe et al. 2007).

### Characterization of dead NRCA8 biomass before and after bioadsorption of Pb^2+^, Ni^2+^, Mn^2+^ and Zn^2+^ by SEM- EDX and FTIR spectroscopy

**Fig. 7:**
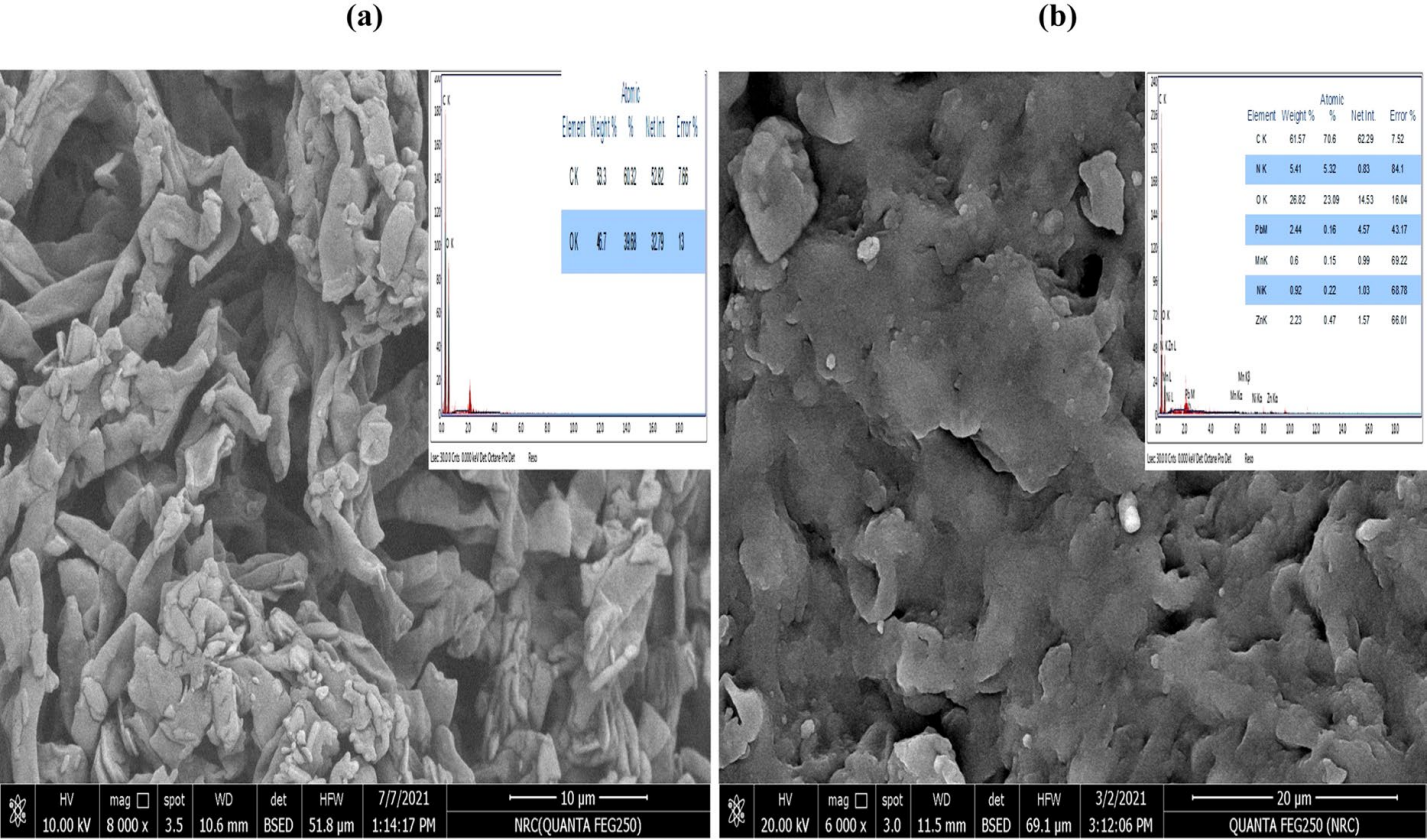
The SEM and EDX analysis of the surface morphology of dead NRCA8 biomass (**a**); before and (**b**); after adsorption

**Fig. 8:**
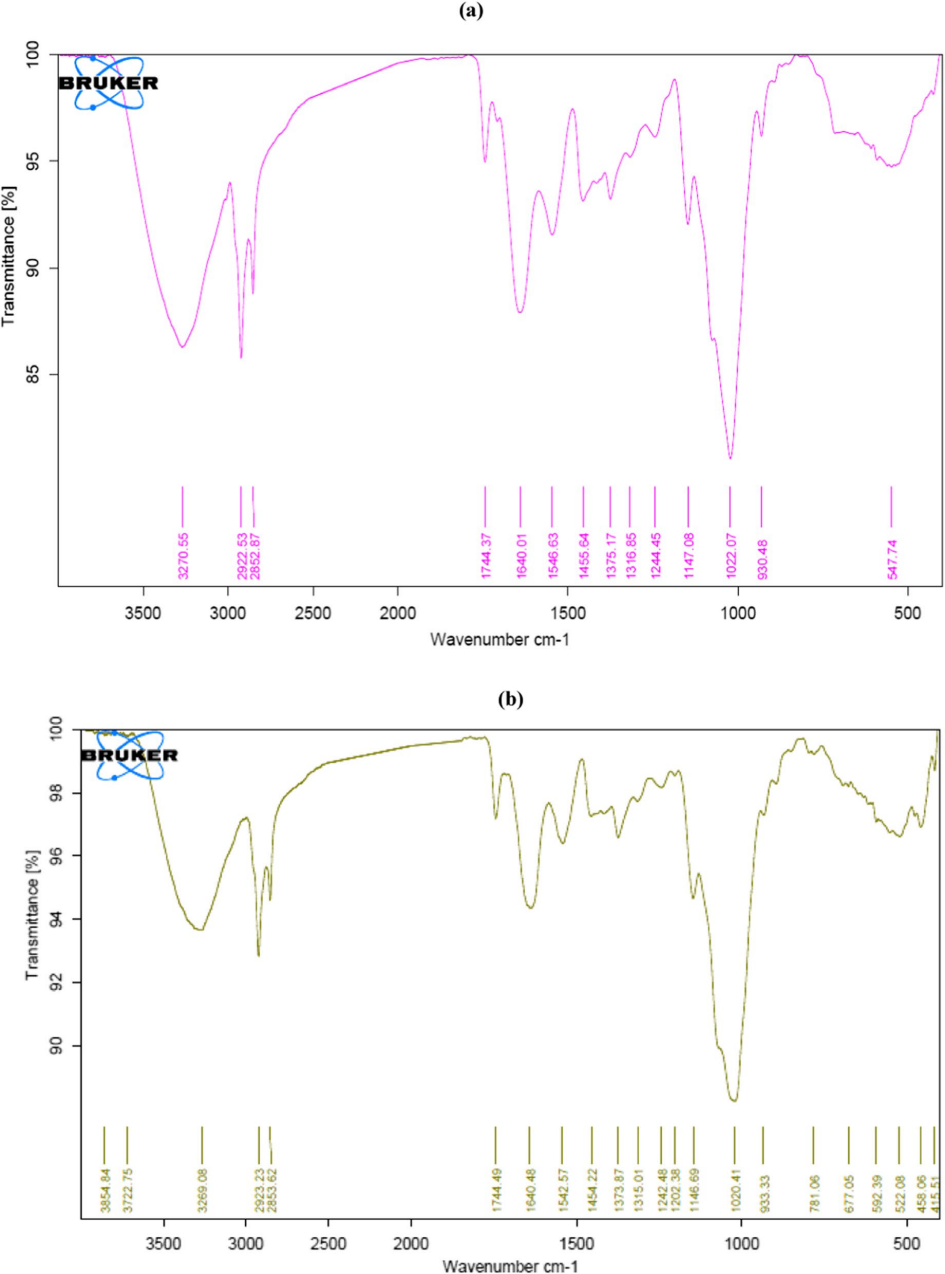
The FTIR analysis of the unloaded (**a**) and loaded (**b**) dead NRCA8 biomass before and after adsorption

The SEM–EDX and IR analyzes are appeared in Figs. 7a, b and 8a, b; respectively. The SEM analysis in Fig. 7a and b showed the surface morphology of untreated and treated dead NRCA8 biomass. The unloaded NRCA8 dead biomass displayed the incidence of distinct, regular, intact long rod, cylindrical sheets or even ribbon shaped mycelial fibers which were extremely branched and tangled as well as some few uniform conical spiral like structures were observed (Fig. 7a). Furthermore, unloaded NRCA8 dead biomass included huge numbers of unoccupied pores with heterogeneous aggregation of single particles (Fig. 7a). However, the SEM image upon treatment the biomass of *Cladosporium* sp. NRCA8 biomass with the heavy metals Pb^2+^, Ni^2+^, Mn^2+^ and Zn^2**+**^, from the multi-metals aqueous solution under the optimized conditions (Fig. 7b) showed blocking of the vacant sites, representing the connection of metal ions onto the surface of NRCA8 dead biomass. Conversely, significant morphological variations were detected in the hyphal shape including distorted, shrunken and irregular expansion of thick fungal mycelial mass with some broad conical deformations in the existing of various heavy metals (Fig. 7b). These changes after treatment might be because of the uptake and accumulation of metal ions lead to variations in physiological, morphological, cellular and molecular levels as well as stress conditions that cause an increase in the area of interaction of metal ions with the fungal biomass. Morphological variations in the fungal mycelia below heavy metals stress have been stated previously (Liaquat et al. 2020), which might be because of oxidation of protein and DNA molecules, variations in ultrastructure, or inhibition of antioxidant defense system in cells (Chen et al. 2014). Bankar et al. (2018) reported that a study of the dimorphism on the marine yeast *Yarrowia* *lipolytica* revealed a change in morphology as elongated, oval or rounded in reaction to various heavy metals stress containing Pb^2**+**^ and Cd^2**+**^.

Furthermore, the EDX was applied to know the chemical composition of NRCA8 dead biomass before and following the uptake of Pb^2+^, Ni^2+^, Mn^2+^ and Zn^2+^ (Fig. 7a, b). The elemental profile of EDX indicated that the C and O contents were 53.3% and 46.7% in terms of weight (Fig. 7a). It was observed that the detectable amounts of N^3+^, C^4+^, O^2+^, Pb^2+^, Mn^2+^, Ni^2+^ and Zn^2+^ were detected to be adsorbed on the fungal mycelia after the handling with multi-metals solution and metals blend (Fig. 7b). A noticeable peak of Pb^2+^, Mn^2+^ and Zn^2+^ adsorbed by the cell wall fungus was detected in the EDX. Conversely, there were no important peaks for the Ni^2+^ and Mn^2+^ were existing viewing an intracellular accumulation of these metals rather than binding on the cell surface (Fig. 7b). Also, laser scanning microscopy images proposed that an intracellular localization primarily within vacuoles and vesicles (Sharma et al. 2022; Traxler et al. 2022). Interestingly, after treatment with Pb^2+^, Zn^2+^, Ni^2+^ and Mn^2+^; it is evident from Fig. 7b that the amount of these ions on the surface increases significantly from 0.0% in the untreated sample to 2.44, 2.23, 0.92 and 0.60, respectively in the multimetals-treated sample. The amount of O (II) decreases from 46.7 to 26.82% while the amount of C and N was increased from 53.3 and 0.0% in untreated sample to 61.57% and 5.41%, respectively in loaded NRCA8 sample (Fig. 7b).

FTIR analysis on fungal biomass functional groups in Fig. 8a and b illustrations the FTIR spectra of untreated and treated dead NRCA8 biomass achieved with Pb^2+^, Ni^2+^, Mn^2+^ and Zn^2+^ adsorption experiments. Data in Fig. 8a of the unloaded-NRCA8 biomass before the adsorption process showed distinct beaks at 3270.55, 2922.53, 2852.87, 1744.37, 1640.01, 1546.63, 1455.64, 1375.17, 1316.85, 1244.45, 1147.08, 1022.07, and 930.48 cm^−1^, respectively. After treatment these peaks were shifted to 3269.08, 2923.23, 2853.62, 1744.49, 1640.48, 1542.57, 1454.22, 1373.87, 1315.01, 1242.48, 1146.69, 1020.41, and 933.33 cm^−1^ (Fig. 8b). The strong, broadband around 3550–3200 cm^−1^ can be allocated to bind alcohol O–H stretching with pb^2+^, Ni^2+^, Zn^2+^ and / or Mn^2+^ while the sharp peaks around 2923, and 2853.62 cm^−1^ can be assigned to N–H stretching binding with these metals (3000–2800 cm^−1^, Fig. 8b). Moreover, the characteristic peaks at 1744.37 (1750–1735 cm^−1^), 1640.48 (1690–1615 cm^−1^), 1542.57 (1590–1500 cm^−1^), 1454.22, (1454–1420 cm^−1^), 1373.87, (1400–1350 cm^−1^), and 1315.01 cm^−1^ refer to strong C=O stretching ester, N–H deformation of amide I band and C=O stretching, strong N–O stretching, medium CH3 bending, alcohol O–H bending, strong C–N stretching aromatic amine (1342–1266 cm^−1^) and the small peaks at 1242.48, 1202.38, and 1146.69 cm^−1^ indicated the strong C-O stretching binding to the heavy metals under study (Fig. 8b). Moreover, a large distinictive peak was detected at 1020.41 cm^−1^ (C=C, C–C, C–O–P and C–O–C groups of saccharides) as well as at 933.33 cm^−1^ (C–O stretching vibration) with Pb^2+^, Zn^2+^, Ni^2+^ and Mn^2+^ (Fig. 8b). After adsorption, new characteristic peaks were generated in the NRCA8-loaded biomass compared to the untreated biomass. These new characteristics bands were detected at 3854.84, 3722.75, 781.06, 677.05, 592.39, 522.08, 458.06, and 415.51 cm^−1^ while the beak at 547.74 cm^−1^ in the untreated-biomass was disappeared after the treatment (Fig. 8a, b). New adsorption peaks at 3854.84 cm^−1^ indicate strong water OH stretch due to surface adsorbed water while a broad adsorption peak at 3722.75 cm^−1^ indicates the medium and sharp alcohol O–H stretching, at 781.06 cm^−1^ mention to strong C–Cl, bands at 677.05 cm^−1^ mention to strong alkene C=C bending, at 592.39 cm^−1^ are indicators for the presence of strong C–Br but peaks at ~ 500 to ~ 400 cm^−1^ coild be strong C–I (Fig. 8b). El-Gendy et al. (2017a) described that the category of functional groups existing depended on the species of fungal. Consistent with our results, representative infrared peaks of un-treated and treated *T.* *brevicompactum* QYCD-6 with metals mixture (Cu, Cr, Cd, Pb and Zn) showed the implicated functional groups of fungal biomasses involved amino, hydroxyl, carbonyl, phosphoryl, nitro and other groups as well as the involvement of biosorption for metals removal (Zhang et al. 2020). Furthermore, Cd uptake by *P.* *chrysosporium* is accredited to hydroxyl, carboxylic and amino functional groups (Noormohamadi et al. 2019) as well as hydroxyl, ethers, amines / amides, carboxylic acid and phosphatidate groups of *P.* *chrysogenum* CS1 were complicated in Cr and Pb adsorption (Qian et al. 2017) but hydroxyl, amides, carboxyl and sulfhydryl groups of *P.* *ostreatus* ISS-1 were drawn into Pb adsorption (Wang et al. 2019).

### Application of biosorption for wastewater treatment by NRCA8 dead biomass

The World Health Organization (WHO) standard for the highest and maximum permissible limits, respectively of heavy metals in wastewater in mg/L such as Fe^3+^ (1.0, and 3.0), Cu^2+^ (0.5, and 2.0), Zn^2+^ (1.0, and 3.0), Pb^2+^ (0.01, and 0.4), Ni^2+^ (0.01, and 0.02), Cr^6+^ (0.05), Cd^2+^ (0.003, and 0.03), Mn^2+^ (0.4), Hg^+^ (0.001), Al^3+^ (0.05 -0.20), and Co^2+^ (0.01) were shown in Table 7 according to the literatures (Musa et al. 2013; Kinuthia et al. 2020). By comparing the heavy metals concentration values detected in the fertilizer industrial effluent with the standard ranges set by the WHO, we find out that all heavy metals detected including Fe^3+^, Cu^2+^, Zn^2+^, Pb^2+^, Ni^2+^, Cr^6+^, Cd^2+^, Hg^+^, Mn^2+^, Al^3+^, and Co^2+^ (50.80, 33.58, 43.61, 9.74, 3.69, 0.29, 0.82, 1.18, 6.19, 2.51, and 0.88 mg/L) were much higher above the permissible limits of WHO standards (Table 7). Heavy metals present in industrial wastewater is the major environmental exertion owing to its toxicity and accumulative in food chain because they are non-biodegradable and then heavy metals removal from industrial effluents and aqueous solutions is of high attention due to the vast quantity of wastewater released into the environment (Sumalatha et al. 2022b). The data in Table 7 indicated that dead NRCA8 biomass was an appropriate tool to treat with the coexistence of different kinds of metallic ions including Cr^6+^, Cd^2+^, Pb^2+^, Hg^+^, Cu^2+^, Ni^2+^, Mn^2+^, Zn^2+^, Fe^3+^, Al^3+^ and Co^2+^ in fertilizer industrial wastewater. NRCA8 dead biomass was able to reduce the ions Cr^6+^, Cd^2+^, Pb^2+^, Ag^+^, Cu^2+^, Ni^2+^, Mn^2+^, Zn^2+^, Fe^3+^, Al^3+^ and Co^2+^ in real industrial wastewater to 0.01, 0.14, 0.00, 0.00, 4.19, 0.05, 0.00, 0.00, 2.33, 0.00, and 0.04 mg/L, respectively (Table 7). The data in Table 7 showed that in the real industrial wastewater containing multi-metals, the dead biomass of NRCA8 showed the highest affinity towards Pb^2+^, Hg^+^, Mn^2+^, Zn^2+^ and Al^3+^ (RE = 100%) ˃ Ni^2+^ (RE = 98.65%) ˃ Cr^6+^ (RE = 96.55%) ˃ Co^2+^ (RE = 95.46%) ˃ Fe^3+^ (RE = 95.41%) ˃ Cu^2+^ (RE = 87.62%) ˃ Cd^2+^ (RE = 82.93%). Sharma et al. (2022) reported that the bioremediation of industrial wastewater by *P.* *chrysosporium*, *P.* *brevispora* and *P.* *floridensis* is a new efficient and eco-friendly method as it exposed a maximum removal of 99–98% for nickel, 98–97% for cadmium, and 12–98% for lead from the industrial wastewater. Furthermore, NRCA8 dead biomass was able to reduce pH, turbidity, TSS, TDS, oil and grease, COD, nitrogen and phosphorus by 14.65%, 77.22%, 89.80%, 83.33%, 50.0%, 68.29%, 88.34% and 78.98%, respectively while it increased the temperature by 16.36% (Table 7). Shah and Rodriguez-Couto (2022) reported that development in wastewater treatment research and processes depending mainly on the fungal method covers the active and applicable role that fungi play in the degradation of xenobiotic compounds, bioremediation of metals mediated, decolorization, bioremediation of petroleum and aromatic hydrocarbons of industrial effluents.

**Table 7:** Assesment of fertilizer industrial effluent composition before and after adsorption by NRCA8 dead biomass

Parameter*	Raw	Treated %	Reduction (R%)	Egypt law	USEPA	WHO
pH	7.92	6.76	14.65	6.0–9.5	NM	NM
Temperature turbidity	27.50	32.00	− 16.36	43.00	NM	NM
TSS	18.26	4.16	77.22	NM	NM	NM
TDS	68.52	6.99	89.80	8.00	NM	NM
Oil and grease	600.33	100.00	83.33	97.00	NM	NM
COD	0.62	0.31	50.00	100.00	NM	NM
Nitrogen Phosphorus	123.00	39.00	68.29	11.00	NM	NM
Cr^6+^	190.00	22.16	88.34	100.00	NM	NM
Cd^2+^	48.72	10.24	78.98	25.00	NM	NM
Pb^2+^	0.29	0.01	96.55	0.50	0.10	0.05–0.05
Hg^+^	0.82	0.14	82.93	0.20	0.005	0.003–0.03
Cu^2+^	9.74	0.00	100.00	1.00	0.015	0.40–0.40
Ni^2+^	1.18	0.00	100.00	0.005	0.001	0.001–0.001
Mn^2+^	33.58	4.19	87.62	1.50	1.30	0.5–2.00
Zn^2+^	3.69	0.05	98.65	1.00	NM	0.01–0.02
Fe^3+^	6.19	0.00	100.00	NM	0.05	0.40–0.40
Al^3+^	43.61	0.00	100.00	NM	5.00	1.00–3.00
Co^2+^	50.80	2.33	95.41	NM	0.30	1.00–3.00
	2.51	0.00	100.00	NM	0.05–0.20	0.05–0.20
	0.88	0.04	95.46	NM	NM	0.01–0.01

*All determents in mg/L, pH in pH units, temperature in °C, and turbidity in NTU

*NM* not mentioned

## Conclusions

The results of this work indicate toward the onsite use dead fungal biomass of the multi-metals hyper tolerant fungus *Cladosporium* sp. NRCA8 isolated from the mycobiome of the fertilizer industry effluents in the multi-adsorption of Pb^2+^, Ni^2+^, Zn^2+^ and Mn^2+^ from multi-metals aqueous solutions and industrial wastewater. The highest multi- adsorption of these heavy metals was achieved by fungus NRCA8 after optimizing operating conditions including temperature; 30 °C, pH; 5.5–6.0, interaction period; 30 min, biosorbent dose; 5.0 g/L for uptake and removal of each ion for removal and uptake capacity and agitation speed; 150 rpm, respectively. Moreover, SEM showed the cellular changes of the fungus biomass resulting from the multi-adsorption of these heavy metals. EDX confirmed the involvement of biosorption and intracellular accumulation in the removal and uptake of these ions as well as FTIR showed that the bioremoval and uptake of Pb^2+^, Ni^2+^, Zn^2+^ and Mn^2+^ were attributed to the functional groups of *Cladosporium* sp. NRCA8 biomass included hydroxyl, carbonyl, ethers, amines/amides and amino. The adsorption data between the adsorbate ions (Pb^2+^, Ni^2+^, Zn^2+^ and Mn^2+^) and the adsorbent NRCA8 biomass at equilibrium condition were estimated by various adsorption isotherm systems containing Langmuir, Freundlich and DKR. Since there is no important variance in the R^2^ values for the three isotherm systems for the adsorption of Pb^2+^, Ni^2+^ and Mn^2+^ onto dead NRCA8 biomass, they can follow the DKR, Freundlich and Langmuir models. The DKR and Freundlich isotherms did not give a good fit for Zn^2+^ sorption onto NRCA8 biomass under multi-metals system but well followed the Langmiur isotherm. Under the optimized operating conditions NRCA8 dead biomass proved to be an appropriate tool to treaty with the coexistence of different metallic ions containing Cr^6+^, Cd^2+^, Pb^2+^, Ag^+^, Cu^2+^, Ni^2+^, Mn^2+^, Zn^2+^, Fe^3+^, Al^3+^ and Co^2+^ in industrial effluents. The data provided in the present work could be helpful for further refinement of the bioremediation processes by using mycobiome of contaminated area as a hopeful choice for the remediation of multiple heavy metals contaminated wastewater and other sites.

## Data Availability

All data generated or analyzed during this study are included in this published article.
